# Risk stratification for adjuvant radiotherapy in pathologic T3N0 rectal cancer using a DeepSurv-based survival model

**DOI:** 10.3389/fonc.2026.1828958

**Published:** 2026-06-03

**Authors:** Yunxia Huang, Yanzong Lin, Qingyang Zhuang, Xinyuan Wu, Qiping Fan, Junxin Wu

**Affiliations:** 1 Department of Radiation Oncology, Clinical Oncology School of Fujian Medical University, Fujian Cancer Hospital, Fuzhou, China; 2 Department of Radiation Oncology, The First Affiliated Hospital of Xiamen University, School of Medicine, Xiamen University, Teaching Hospital of Fujian Medical University, Xiamen, China; 3 Department of General Surgery, The First Affiliated Hospital of Xiamen University, School of Medicine, Xiamen University, Teaching Hospital of Fujian Medical University, Xiamen, China; 4 Department of Urology, The First Clinical Medical College of Southern Medical University, Guangzhou, China; 5 Department of Public Health Sciences, Clemson University, Clemson, SC, United States

**Keywords:** adjuvant radiotherapy, DeepSurv, pT3N0 rectal cancer, risk stratification, survival model

## Abstract

**Background:**

The role of adjuvant radiotherapy in patients with pathologic T3N0 rectal cancer remains controversial. Although overall prognosis is favorable, marked heterogeneity exists, and reliable tools to identify patients who may derive meaningful benefit from postoperative radiotherapy are lacking. This study aimed to develop and validate a deep learning-based prognostic model to support individualized radiotherapy decision-making in this population.

**Methods:**

We included 1,411 patients with pT3N0 rectal adenocarcinoma from the Surveillance, Epidemiology, and End Results (SEER) database for model development and internal validation. An independent real-world cohort of 118 patients was used for external validation. A DeepSurv-based survival model was constructed using clinicopathological variables to predict cancer-specific survival (CSS). Model performance was evaluated using time-dependent area under the receiver operating characteristic curve (AUC). SHapley Additive exPlanations (SHAP) were applied to improve model interpretability. Subgroup analyses were conducted to assess the survival impact of adjuvant radiotherapy across model-defined risk strata.

**Results:**

The DeepSurv model showed good discrimination, with 3-, 5-, and 10-year AUCs of 0.749, 0.739, and 0.769 in the training set and 0.819, 0.753, and 0.711 in the external validation cohort, respectively. Using the prespecified cutoff, risk stratification revealed differential survival patterns according to adjuvant radiotherapy status. Specifically, adjuvant radiotherapy was associated with improved survival in high-risk patients in both the SEER cohort (log-rank P < 0.001) and the external cohort (log-rank P = 0.032), but not in low-risk patients. Building on our prior Cox-based approach, DeepSurv captured nonlinear relationships and interactions to better individualize radiotherapy benefit. SHAP analysis identified carcinoembryonic antigen status as the most influential predictor.

**Conclusions:**

This DeepSurv-based prognostic model effectively stratifies patients with pT3N0 rectal cancer and may help identify individuals who could potentially benefit from adjuvant radiotherapy. A risk-adapted postoperative treatment strategy may support individualized decision-making by prioritizing radiotherapy for high-risk patients while avoiding potential overtreatment in low-risk individuals.

## Introduction

Rectal cancer remains a leading contributor to the global cancer burden (1), and multimodal therapy has substantially improved survival outcomes over recent decades. Contemporary clinical practice integrates high-quality total mesorectal excision (TME) with appropriate systemic and local therapies to maximize both local and distant disease control (2). However, postoperative decision-making for pathologic T3N0 (pT3N0) rectal cancer remains highly individualized and controversial due to marked prognostic heterogeneity, highlighting the need for robust and data-driven risk stratification models to identify patients who may derive meaningful benefit from adjuvant radiotherapy.

Current guideline recommendations mirror this clinical ambiguity. The National Comprehensive Cancer Network (NCCN) Clinical Practice Guidelines in Oncology for Rectal Cancer acknowledge that adjuvant radiotherapy may be considered in select patients with high-risk pathologic Stage II (such as pT3N0), particularly if neoadjuvant therapy was not administered, but there is no uniform mandate for all pT3N0 patients. Observation following high-quality TME is considered an acceptable option for those deemed at lower risk. The NCCN further emphasizes that postoperative radiotherapy should be individualized based on adverse pathologic features, such as threatened circumferential resection margins or other high-risk characteristics (3). Meanwhile, ESMO guidelines emphasize a personalized, multidisciplinary decision-making approach and recognize that adjuvant radiotherapy is not routinely required for all Stage II rectal cancer, including T3N0 tumors, due to limited evidence demonstrating unequivocal survival benefit in the modern TME era (4). The body of evidence examining adjuvant radiotherapy in pT3N0 rectal cancer is limited and heterogeneous (5). Several retrospective cohort studies and population-based analyses, including those derived from the Surveillance, Epidemiology, and End Results (SEER) database, have reported inconsistent associations between postoperative radiotherapy and survival outcomes, with some suggesting modest improvements in cancer-specific survival (CSS) (6). Nevertheless, the quality of evidence remains low, and no large randomized controlled trials have specifically addressed this question in patients with pT3N0 status treated with upfront surgery alone (7). Some studies evaluating radiotherapy’s effect on locoregional recurrence in T3N0 patients managed with TME found a non-significant trend toward reduced local recurrence with radiotherapy, but the data were derived predominantly from small retrospective studies and lacked prospective validation (7, 8). Importantly, given the generally favorable natural history of pT3N0 rectal cancer following high-quality TME, with 5-year overall survival exceeding 70–80% and low local recurrence rates, routine postoperative radiotherapy may expose a substantial proportion of patients to unnecessary treatment-related morbidity without clear oncologic benefit (9).

Underlying this clinical equipoise is substantial heterogeneity of individual risk within the pT3N0 population. Although outcomes are generally favorable after upfront surgery, a non-negligible subset of patients experiences disease recurrence or cancer-related mortality, underscoring the limitations of uniform postoperative management strategies (10). Traditional prognostic tools often rely on limited clinicopathologic factors and assume linear relationships between variables and outcomes, which may insufficiently capture complex interplays among risk determinants. In contrast, recent advances in survival modeling, particularly deep learning methods such as DeepSurv (11), offer the capability to model nonlinear effects and to generate individualized risk scores for time-to-event outcomes. These approaches have shown promise in oncology for refined risk stratification and potential prediction of treatment benefit (12, 13), but their application to postoperative decision-making in pT3N0 rectal cancer, especially regarding adjuvant radiotherapy, remains underexplored.

To address this gap, we conducted a population-based study using data from the SEER database to develop and internally validate a DeepSurv-based prognostic model for patients with postoperative pT3N0 rectal cancer. Individualized risk scores were generated to stratify patients into clinically distinct high- and low-risk subgroups. Within these strata, we examined the association between adjuvant radiotherapy and survival outcomes. To enhance the clinical relevance of our findings, we further conducted external validation using an independent real-world cohort of 118 patients treated at a tertiary cancer center. We hypothesized that adjuvant radiotherapy would be associated with improved survival among patients classified as high risk, but not among those in the low-risk group, thereby providing a data-driven framework to support individualized postoperative treatment decisions in this clinically challenging population.

## Materials and methods

### Study population and data sources

This study comprised two independent cohorts. The development and internal validation cohort was derived from the SEER database, which collects population-based cancer registry that collects incidence and survival data and covers approximately 28% of the United States population (14).

For development and internal validation, patients diagnosed with rectal adenocarcinoma (International Classification of Diseases for Oncology, Third Edition [ICD-O-3] site code C20.9, Rectum, NOS) between 2010 and 2017 were initially identified from the SEER database. Inclusion criteria for patients were: (1) patients with pathologic stage T3N0M0 disease identified based on staging information available in the SEER database, corresponding to the definition in the 8th edition of the American Joint Committee on Cancer (AJCC). Although AJCC 8th edition terminology was adopted for consistency with current clinical practice, staging information in the SEER database (2010–2017) is based on the AJCC 7th edition. Given that the definition of pT3N0M0 disease remains unchanged between the 7th and 8th editions, no additional conversion was required; (2) receipt of upfront curative-intent resection without any neoadjuvant therapy; and (3) availability of complete clinicopathologic and survival information. A total of 2,954 patients met the initial inclusion criteria.

**Figure 1:**
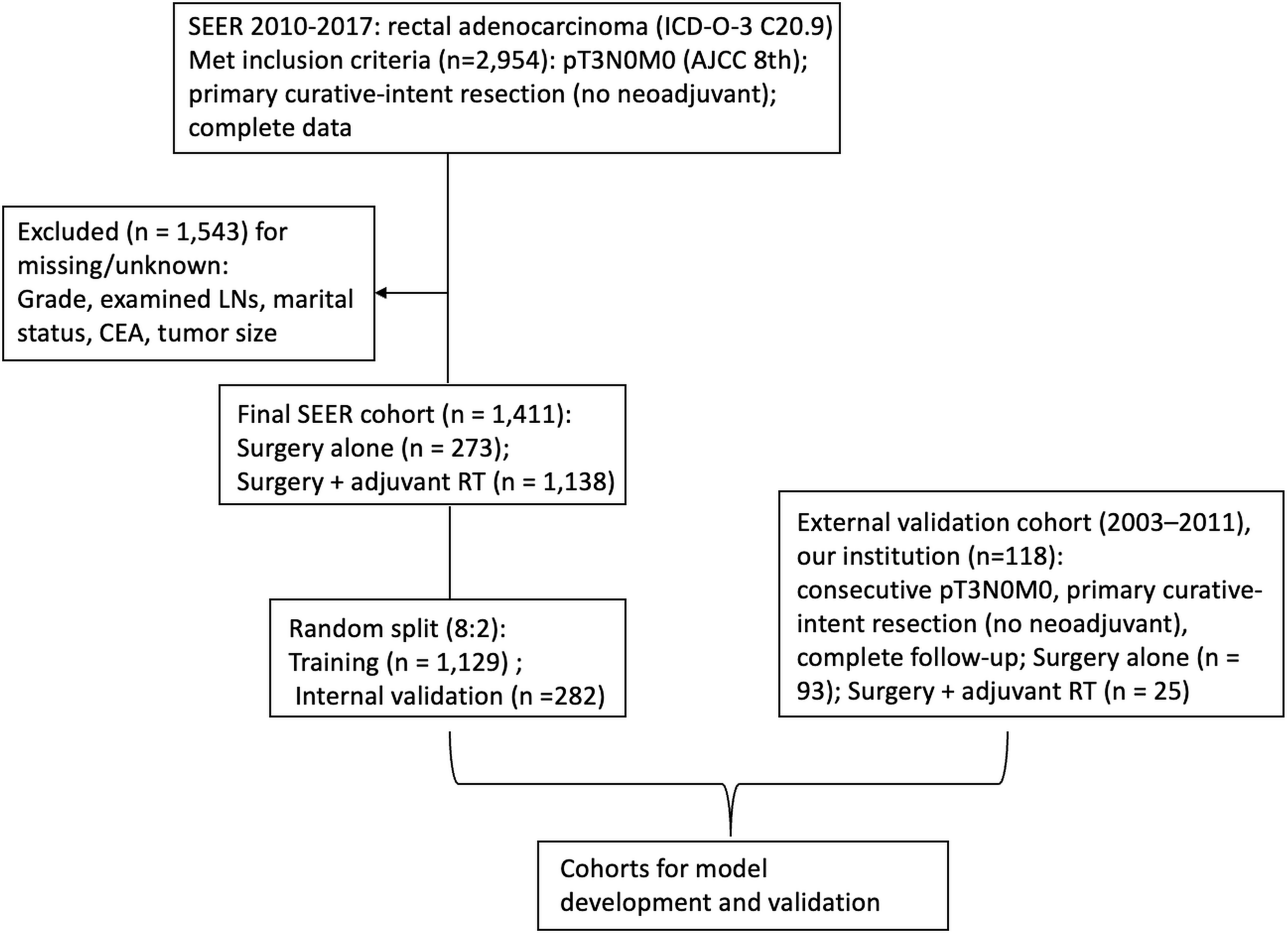
Flowchart of patient selection and cohort construction.

Patients were subsequently excluded if they had missing or unknown information on tumor grade (n = 224), number of lymph nodes examined (n = 11), marital status (n = 123), carcinoembryonic antigen (CEA) status (n = 903), or tumor size (n = 912). The patient selection process is illustrated in **Figure 1**. After applying these exclusion criteria, 1,411 patients were included in the final SEER cohort. Among them, 1,138 patients received adjuvant radiotherapy, whereas 273 did not receive adjuvant radiotherapy. According to combined radiotherapy and chemotherapy status, 201 patients underwent surgery only, 72 received chemotherapy only, 31 received radiotherapy only, and 1,107 received chemoradiotherapy.

For external validation, an independent real-world cohort of 118 consecutive patients with pT3N0M0 rectal cancer was retrospectively collected from The Fujian Cancer Hospital between 2003 and 2011, with approval from the institutional ethics committee (Approval No. K2022-124-01). The study period was restricted to 2003–2011 to ensure sufficient follow-up duration and completeness of survival data, which are important for robust external validation. Furthermore, this time frame captured a period of consistent institutional treatment protocols. All patients in the external cohort underwent upfront surgical resection without neoadjuvant therapy and had complete survival follow-up information available. Of these patients, 25 received adjuvant radiotherapy, whereas 93 did not. According to combined radiotherapy and chemotherapy status, 28 patients underwent surgery only, 65 received chemotherapy only, 1 received radiotherapy only, and 24 received chemoradiotherapy.

### Outcome definition

In the SEER cohort, the primary endpoint was cancer-specific survival (CSS), defined as the interval from surgery to death due to rectal cancer, with deaths from other causes censored. CSS was selected to minimize competing-risk bias in population-based registry data. In the external cohort, overall survival (OS) was used as the primary endpoint owing to the reliability of all-cause mortality data.

### Model development and internal validation

A DeepSurv-based survival model was developed in the SEER cohort to estimate individualized relative risk for CSS. Prespecified predictors available at surgery included sex, age, marital status, histologic grade, examined lymph nodes (<12 vs ≥12), carcinoembryonic antigen (CEA) status, tumor size (<5 cm vs ≥5 cm), radiotherapy (yes/no), and chemotherapy (yes/no). Candidate variables were selected based on clinical relevance, data completeness, and availability across both the SEER cohort and the external validation cohort to ensure model transportability and enable direct external validation using an identical feature set. Binary variables were coded as 0/1; categorical variables were one-hot encoded; and continuous variables were standardized using training-set statistics to prevent data leakage.

The SEER cohort was randomly split into a training set (80%; n=1,129) and an internal validation set (20%; n=282). As no off-the-shelf implementation fully met study requirements, DeepSurv was implemented in-house in Python. The network comprised five fully connected hidden layers plus an output layer (nodes: 9, 128, 128, 32, 32, 1), with GELU activations, layer normalization, and no dropout (dropout=0). A linear output layer was used to maintain compatibility with the Cox formulation. Training minimized the negative Cox partial log-likelihood (pycox) and used AdamW (initial learning rate 1×10⁻³) with cosine annealing learning-rate scheduling (CosineAnnealingLR). Training used the full training cohort as a single batch for up to 1,000 epochs, with early stopping based on the minimum validation loss.

Model discrimination in the internal validation set was assessed using Harrell’s concordance index (C-index) and time-dependent ROC at 3, 5, and 10 years. Patients were stratified into high- and low-risk groups using DeepSurv risk scores; the optimal cutoff was determined in the training set via the Youden index and applied unchanged to both the internal validation and external cohorts. Kaplan–Meier analyses with log-rank tests compared survival between risk strata. Within each stratum, the association between adjuvant radiotherapy and survival was evaluated using Cox proportional hazards models, with hazard ratios (HRs) and 95% confidence intervals reported.

To evaluate the added value of the DeepSurv model, a conventional Cox proportional hazards model was constructed using the same clinicopathological variables, including sex, age, marital status, tumor grade, number of lymph nodes examined, CEA status, tumor size, radiotherapy, and chemotherapy. Model performance was assessed using Harrell’s concordance index (C-index) and time-dependent AUC at 3, 5, and 10 years. The discriminative performance of the Cox model was compared with that of the DeepSurv model. For the primary discrimination metrics, including Harrell’s C-index and time-dependent AUCs at 3, 5, and 10 years, 95% confidence intervals were estimated using bootstrap resampling with 1,000 repetitions within each cohort. Time-dependent AUC curves from years 1 to 10 were generated to visually compare the discriminative performance of the DeepSurv and Cox models across follow-up time. Decision curve analysis (DCA) was further performed at 3, 5, and 10 years to compare the clinical net benefit of the two models across different threshold probabilities. In this context, DCA was used to evaluate the potential clinical utility of model-based risk stratification for supporting adjuvant radiotherapy decision-making rather than to establish causal treatment benefit. Model fit of the Cox model was additionally summarized using the partial log-likelihood and Akaike information criterion (AIC). The Cox model was also used to provide conventional feature-level interpretation by reporting hazard ratios (HRs) and 95% confidence intervals (CIs) for each included variable.

Adjuvant treatment modalities were categorized according to radiotherapy and chemotherapy status as surgery alone, chemotherapy alone, radiotherapy alone, or chemoradiotherapy. Surgery alone was defined as no recorded adjuvant radiotherapy or chemotherapy, and chemoradiotherapy as receipt of both treatments. Cox analyses were performed to evaluate the associations of chemotherapy and chemoradiotherapy with survival. These analyses should be considered exploratory because of the imbalanced treatment subgroup distributions, particularly in the external validation cohort.

### External validation

External validation applied the pretrained model to an independent real-world cohort without refitting, using the same model parameters and SEER-derived risk cutoff. Because endpoints differed by data source, validation emphasized risk stratification rather than absolute calibration, with CSS evaluated in SEER and OS evaluated in the external cohort due to endpoint availability.

### Model interpretability

Interpretability was assessed using SHapley Additive exPlanations (SHAP). SHAP values were computed with DeepExplainer using the training dataset as the background reference, and global feature importance was summarized.

### Software and reproducibility

All analyses were performed in Python. The DeepSurv model was implemented in-house and the Cox partial likelihood loss was computed using *pycox* package. Reproducibility and leakage preventaion were ensured by performing preprocessing steps, including standardization and cutoff selection, using the training set only.

## Results

### Patient characteristics and cohort composition

Finally, 1,411 patients with pathologically confirmed pT3N0M0 rectal adenocarcinoma from the SEER database were included for model development and internal validation. Patients were randomly assigned to a training cohort (80%) and an internal validation cohort (20%). An independent cohort of 118 consecutive patients served as the external validation set. Baseline clinicopathological characteristics of patients in each cohort are summarized in **Table 1**.

**Table 1:** Baseline clinicopathological characteristics of patients in each cohort.

Characteristic	SEER cohort (n = 1411)	External cohort (n = 118)	P value*
Age group			0.008
<70 years	1068 (75.7)	102 (86.4)	
≥70 years	343 (24.3)	16 (13.6)	
Sex			0.510
Male	892 (63.2)	71 (60.2)	
Female	519 (36.8)	47 (39.8)	
Marital status			<0.001
Married	868 (61.5)	111 (94.1)	
Unmarried	543 (38.5)	7 (5.9)	
Tumor grade			0.031
Grade I/II	1298 (92.0)	115 (97.5)	
Grade III/IV	113 (8.0)	3 (2.5)	
Tumor size			<0.001
<5 cm	1409 (99.9)	59 (50.0)	
≥5 cm	2 (0.1)	59 (50.0)	
Examined lymph nodes			0.004
<12	430 (30.5)	21 (17.8)	
≥12	981 (69.5)	97 (82.2)	
CEA status			0.575
Negative	872 (61.8)	76 (64.4)	
Positive	539 (38.2)	42 (35.6)	
Adjuvant radiotherapy			<0.001
No	273 (19.3)	93 (78.8)	
Yes	1138 (80.7)	25 (21.2)	
Adjuvant chemotherapy			0.024
No	232 (16.4)	29 (24.6)	
Yes	1179 (83.6)	89 (75.4)	

Data are presented as median for continuous variables and n (%) for categorical variables. *P values are descriptive only and not intended for formal statistical comparison between cohorts; Mann–Whitney U test was used for continuous variables and χ² test for categorical variables. CEA, carcinoembryonic antigen.

### Survival outcomes in the overall population

**Figure 2:**
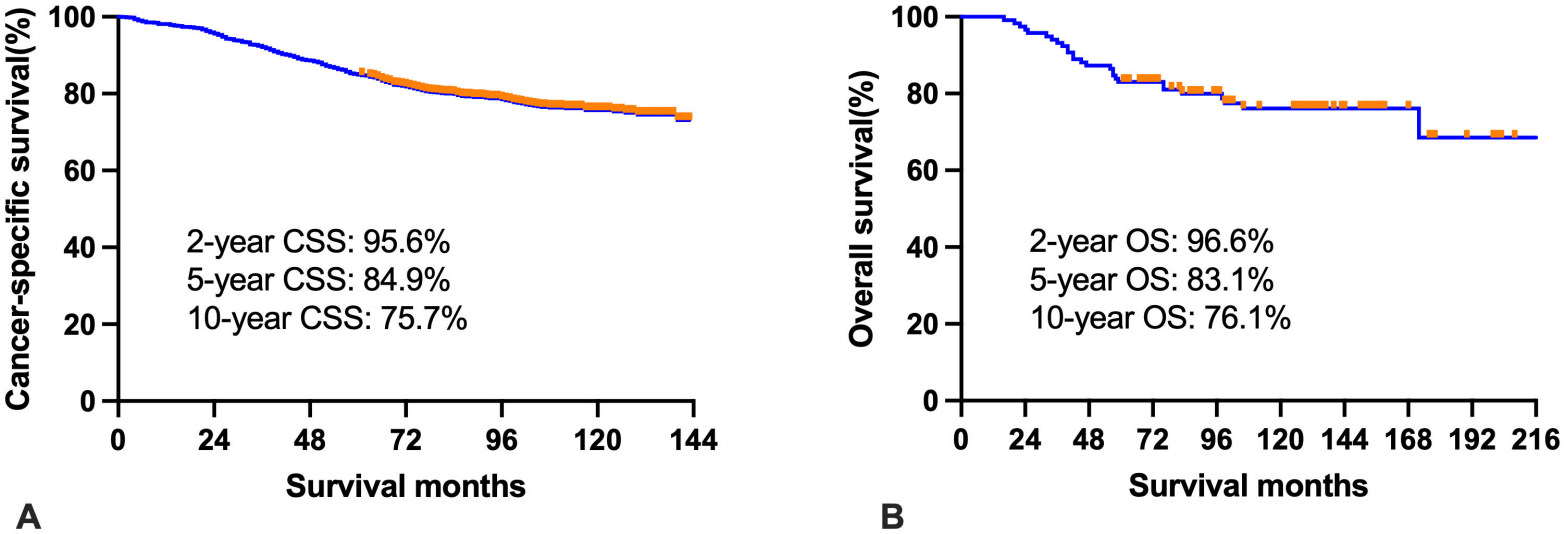
Kaplan–Meier survival curves for the SEER cohort and external validation cohort. **(A)** Cancer-specific survival in the SEER cohort. **(B)** Overall survival in the external validation cohort. The blue step line represents the Kaplan–Meier survival estimate, and the orange tick marks indicate censored observations. CSS, cancer-specific survival; OS, overall survival.

In the SEER cohort, the median follow-up was 92 months (range, 1–143). The 2-, 5-, and 10-year CSS rates were 95.6%, 84.9%, and 75.7%, respectively (**Figure 2**).

In the external validation cohort, the median follow-up was 100 months (range, 6–212), with 2-, 5-, and 10-year overall survival (OS) rates of 96.6%, 83.1%, and 76.1%, respectively (**Figure 2**).

### Performance of the DeepSurv prognostic model

**Figure 3:**
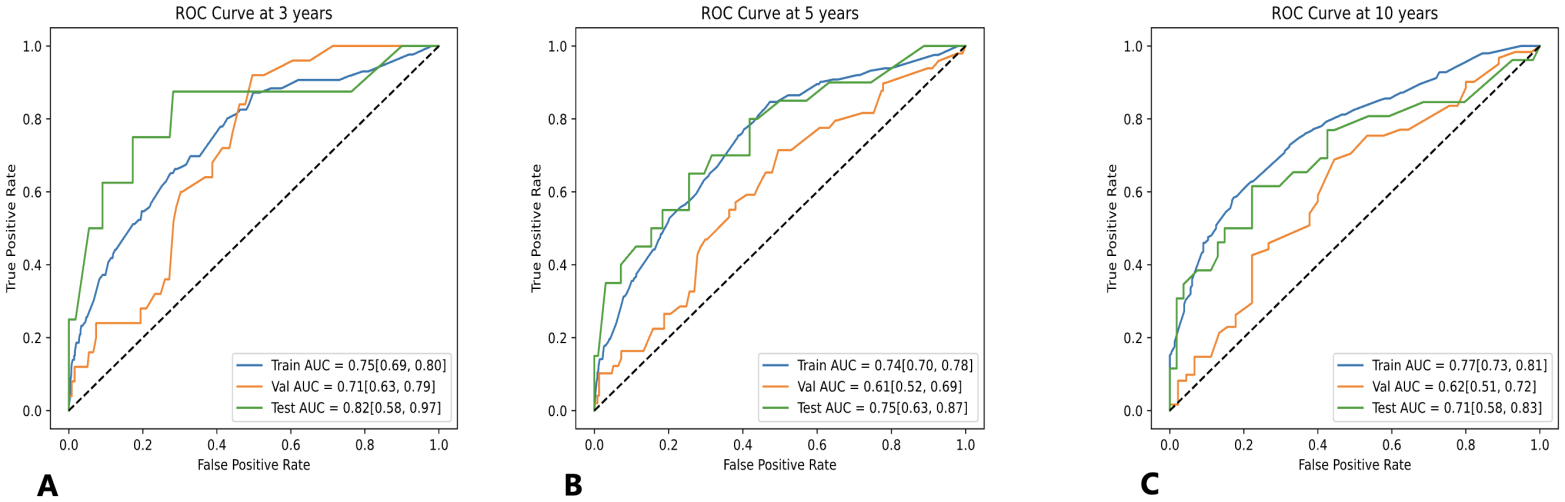
Time-dependent ROC curves of the DeepSurv model in the training, internal validation, and external validation cohorts. Time-dependent receiver operating characteristic (ROC) curves were generated to evaluate the discriminative performance of the DeepSurv-based risk model at **(A)** 3 years, **(B)** 5 years, and **(C)** 10 years. The areas under the curve (AUCs) with 95% confidence intervals are shown for the training set, internal validation set, and external validation cohort. The diagonal dashed line indicates no discrimination.

The DeepSurv model demonstrated good discrimination in predicting survival. Detailed performance estimates with 95% confidence intervals are presented in **Table 2**. In the SEER training set, the 3-, 5-, and 10-year AUCs were 0.749, 0.739, and 0.769, respectively. In the internal validation set, the corresponding AUCs were 0.709, 0.606, and 0.617. In the external validation cohort, the model achieved AUCs of 0.819, 0.753, and 0.711 at 3, 5, and 10 years, respectively (**Figure 3**), suggesting potential generalizability across independent datasets.

**Table 2:** Performance comparison between the DeepSurv model and conventional Cox proportional hazards model.

Model	Cohort	Endpoint	C-index (95% CI)	3-year AUC (95% CI)	5-year AUC (95% CI)	10-year AUC (95% CI)
DeepSurv model	SEER training set	CSS	0.720 (0.689-0.751)	0.749 (0.691-0.802)	0.739 (0.696-0.780)	0.769 (0.727-0.808)
	SEER internal validation set	CSS	0.600 (0.530-0.665)	0.709 (0.628-0.788)	0.606 (0.520-0.685)	0.617 (0.507-0.723)
	External validation cohort	OS	0.711 (0.604-0.810)	0.819 (0.584-0.972)	0.753 (0.632-0.867)	0.711 (0.578-0.831)
Cox PH model	SEER training set	CSS	0.661 (0.628-0.693)	0.692 (0.635-0.747)	0.679 (0.636-0.721)	0.705 (0.656-0.749)
	SEER internal validation set	CSS	0.596 (0.517-0.663)	0.710 (0.597-0.811)	0.591 (0.500-0.676)	0.639 (0.539-0.741)
	External validation cohort	OS	0.661 (0.552-0.773)	0.649 (0.436-0.861)	0.711 (0.584-0.837)	0.661 (0.514-0.798)

C-index indicates Harrell’s concordance index; AUC, area under the time-dependent receiver operating characteristic curve; CSS, cancer-specific survival; OS, overall survival; PH, proportional hazards. The Cox proportional hazards model was constructed using the same clinicopathological variables as the DeepSurv model. CSS was used as the endpoint in the SEER cohort, whereas OS was used in the external validation cohort because cancer-specific survival information was unavailable in the external cohort. The 95% confidence intervals for C-index and time-dependent AUCs were estimated using bootstrap resampling with 1,000 repetitions.

To further assess the added value of the DeepSurv model, we constructed a conventional Cox proportional hazards model using the same clinicopathological variables. Compared with the conventional Cox model, the DeepSurv model showed higher discrimination in the SEER training set and the independent external validation cohort. In particular, in the external validation cohort, the DeepSurv model achieved a higher C-index than the Cox model (0.711 vs. 0.661) and superior 3-, 5-, and 10-year AUCs (0.819 vs. 0.649, 0.753 vs. 0.711, and 0.711 vs. 0.661, respectively). These findings suggest that the DeepSurv model may provide additional prognostic value beyond a conventional linear Cox model.

**Figure 4:**
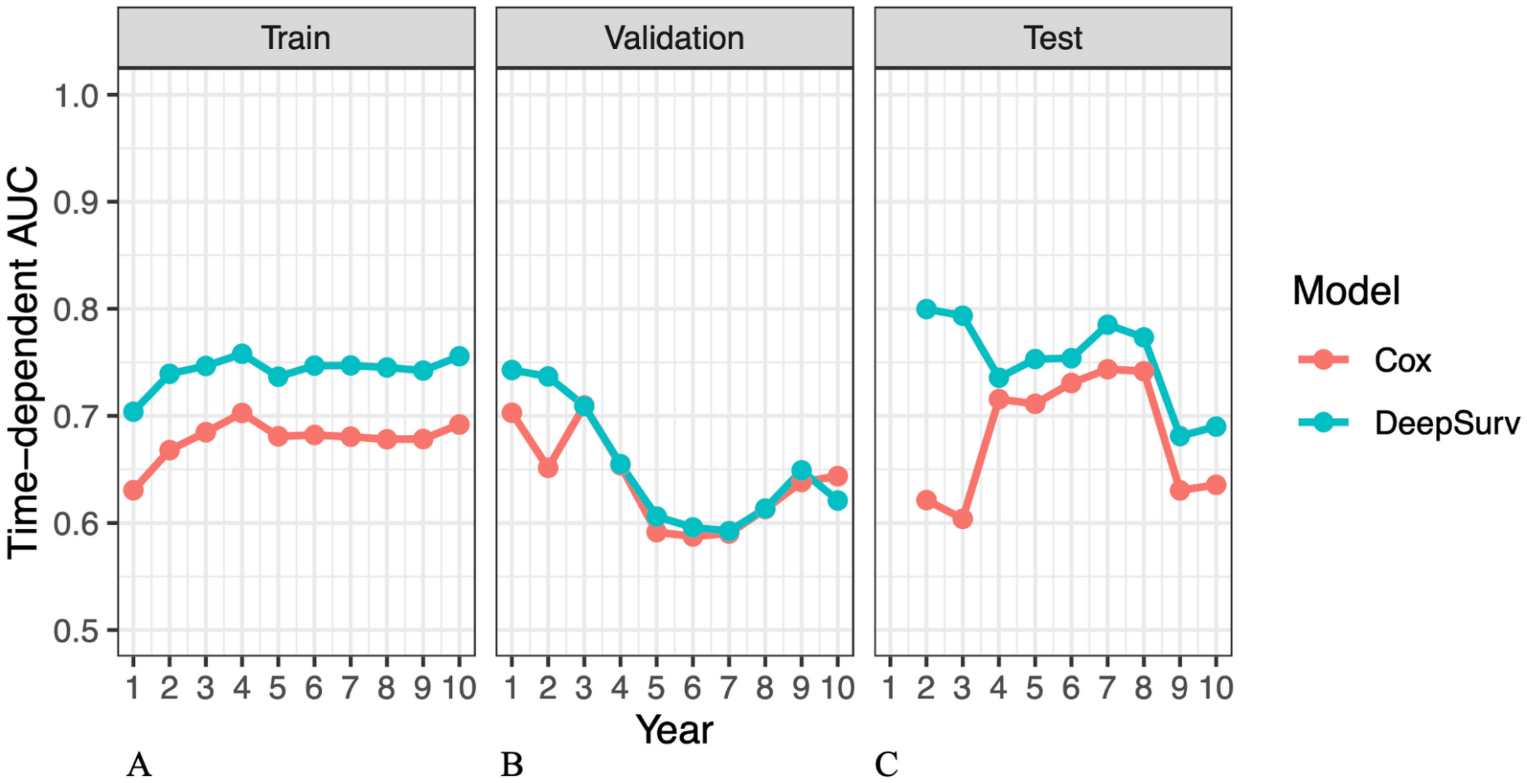
Time-dependent AUC comparison between the DeepSurv model and the conventional Cox proportional hazards model. Time-dependent AUCs from years 1 to 10 are presented for the **(A)** SEER training set, **(B)** SEER internal validation set, and **(C)** external validation cohort. The curves provide a visual comparison of the discriminative performance of the two models across follow-up times. AUC, area under the curve; Cox, Cox proportional hazards model.

Time-dependent AUC curves were generated to visually compare the discriminative performance of the DeepSurv and conventional Cox models across follow-up time(**Figure 4**). The DeepSurv model showed generally higher time-dependent AUCs than the Cox model in the SEER training set and external validation cohort, whereas the differences were less consistent in the internal validation set.

**Figure 5:**
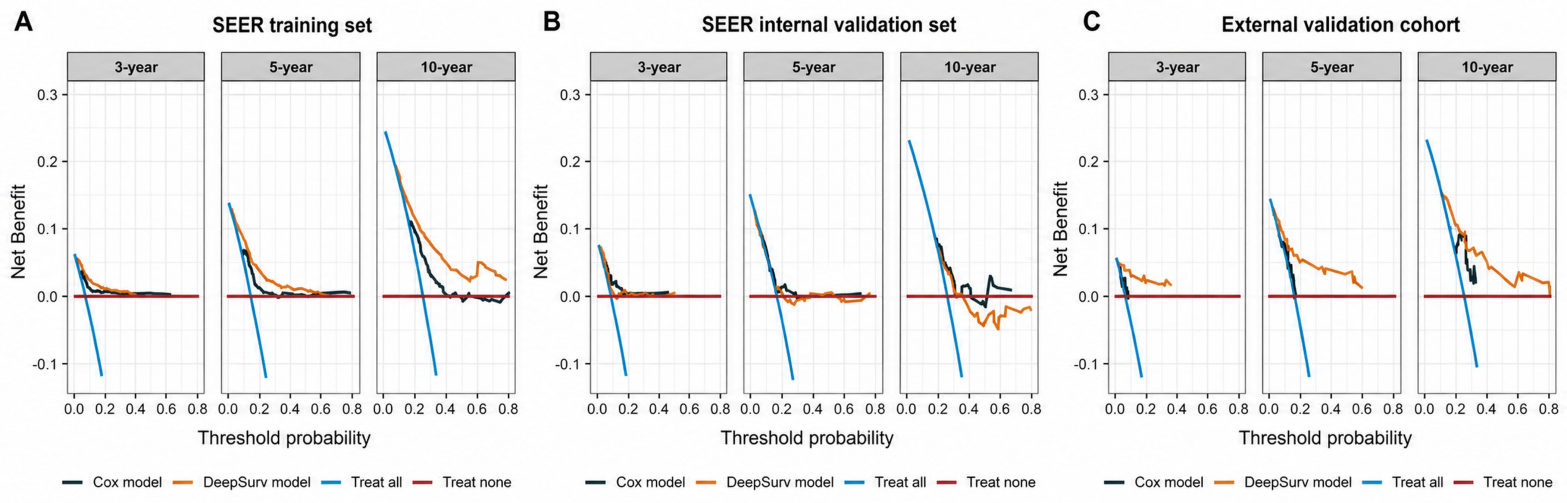
Decision curve analysis comparing the DeepSurv model with the conventional Cox model. Decision curve analyses were performed at 3, 5, and 10 years in the **(A)** SEER training set, **(B)** SEER internal validation set, and **(C)** external validation cohort. The y-axis indicates net benefit, and the x-axis indicates threshold probability. “Treat all” assumes that all patients would be classified as high risk and considered for adjuvant radiotherapy, whereas “treat none” assumes that no patients would be selected. Decision curve analysis was used to assess the potential clinical utility of model-based risk stratification and should not be interpreted as evidence of causal treatment benefit. Cox, Cox proportional hazards model; DCA, decision curve analysis.

Decision curve analysis was further performed to compare the clinical net benefit of the two models at 3, 5, and 10 years(**Figure 5**). The DeepSurv model showed comparable or higher net benefit than the conventional Cox model across clinically relevant threshold probabilities, particularly in the SEER training set and external validation cohort. In the internal validation set, the net benefit of the two models was less consistent, which was in line with the relatively lower discrimination observed in this subset.

In addition, the Cox model provided conventional feature-level interpretation of the included variables. Older age, unmarried status, elevated CEA level, and fewer lymph nodes examined were associated with poorer survival, whereas adjuvant radiotherapy and chemotherapy were not independently associated with survival in the overall SEER cohort(**Table 3**).

**Table 3:** Multivariable cox proportional hazards model for survival in the SEER cohort.

Variable	HR	95% CI
Sex	0.745	0.586–0.946
Age	1.887	1.487–2.396
Marital status	1.455	1.160–1.826
Tumor grade	1.140	0.776–1.674
Lymph nodes examined	0.712	0.565–0.898
CEA status	1.884	1.508–2.354
Tumor size	3.679	0.512–26.413
Radiotherapy	0.988	0.621–1.571
Chemotherapy	0.884	0.546–1.430

HR, hazard ratio; CI, confidence interval; CEA, carcinoembryonic antigen. The Cox proportional hazards model was constructed using the same clinicopathological variables included in the DeepSurv model.

### Differential benefit of adjuvant radiotherapy by risk group

**Figure 6:**
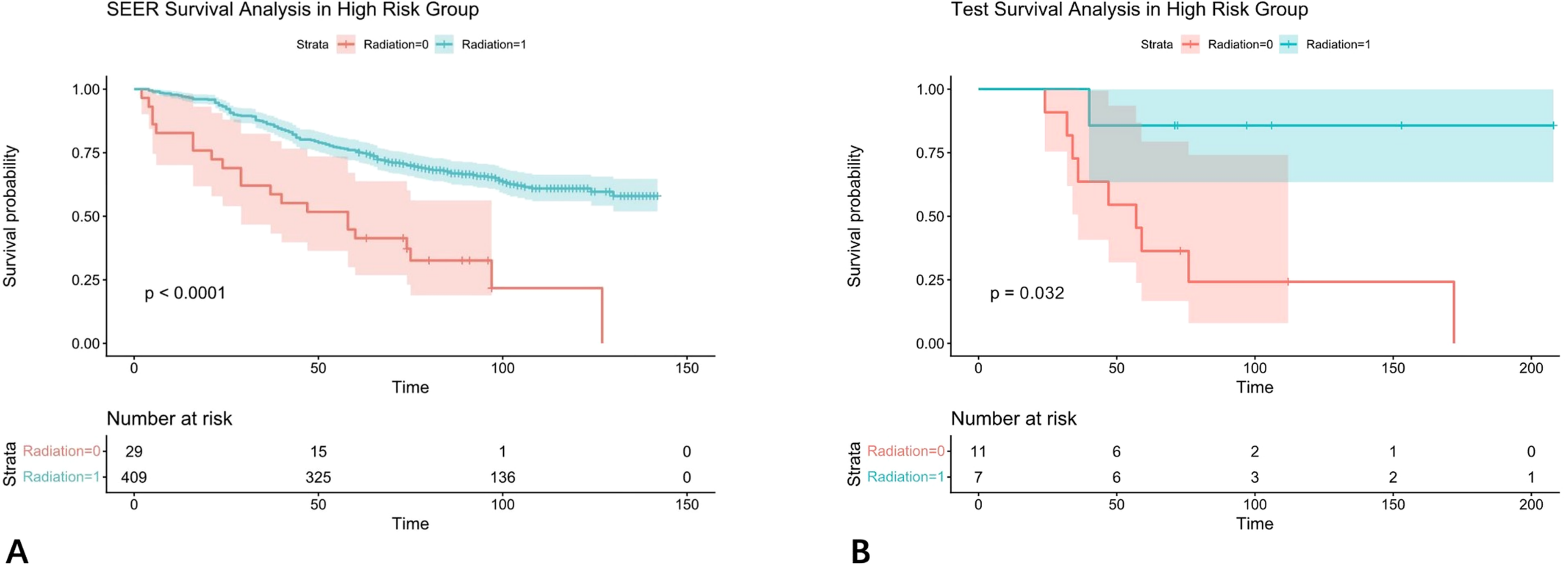
Survival curves comparing patients with and without adjuvant radiotherapy in the high-risk group. **(A)** SEER cohort. **(B)** External validation cohort. The x-axis indicates survival time in months. Shaded areas represent 95% confidence intervals.

**Figure 7:**
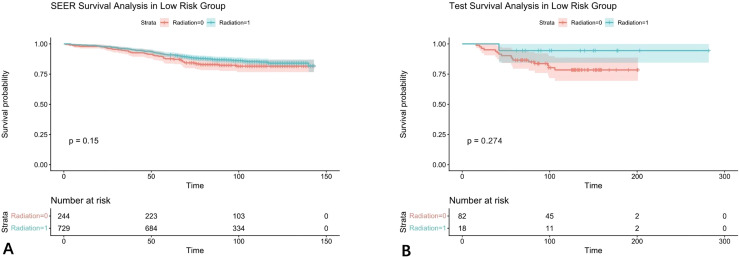
Survival curves comparing patients with and without adjuvant radiotherapy in the low-risk group. **(A)** SEER cohort. **(B)** External validation cohort. The x-axis indicates survival time in months. Shaded areas represent 95% confidence intervals.

Using the prespecified cutoff (0.51), patients were stratified into high- and low-risk groups. This stratification revealed clinically meaningful heterogeneity in treatment effect: adjuvant radiotherapy was associated with improved survival in the high-risk group (**Figure 6**) but not in the low-risk group (**Figure 7**) across both the SEER cohort and the external validation cohort. In the high-risk group, patients receiving adjuvant radiotherapy had significantly better survival than those managed without radiotherapy in the SEER cohort (log-rank P < 0.001) and in the external validation cohort (log-rank P = 0.032) (**Figure 6**). By contrast, among low-risk patients, adjuvant radiotherapy was not associated with a survival benefit in either the SEER cohort (log-rank P = 0.150) or the external validation cohort (log-rank P = 0.274) (**Figure 7**). Stratum-specific hazard ratios are summarized in **Table 4**.

**Table 4:** Radiotherapy benefit by DeepSurv risk strata.

Cohort/endpoint	Risk stratum	RT, n	No RT, n	HR (RT vs no RT) (95% CI)	*P*
SEER/CSS	High risk	409	29	0.310 (0.196, 0.491)	<0.001
	Low risk	729	244	0.772 (0.541, 1.101)	0.15
External/OS	High risk	7	11	0.119 (0.015, 0.944)	0.032
	Low risk	18	82	0.278 (0.037, 2.095)	0.274

Risk strata were defined using the cutoff derived from the training set (Youden index) and applied unchanged to the internal validation and external cohorts.

Endpoints: CSS in SEER; OS in external cohort.

HRs were estimated using Cox models within each risk stratum (RT vs No RT).

### Exploratory analyses of other adjuvant treatment modalities

We further explored survival patterns according to other adjuvant treatment modalities. In the SEER cohort, 201 patients underwent surgery alone, 72 received chemotherapy alone, 31 received radiotherapy alone, and 1,107 received chemoradiotherapy. In the external validation cohort, the corresponding numbers were 28, 65, 1, and 24, respectively.

In exploratory Cox analyses, adjuvant chemotherapy was not significantly associated with survival in either the SEER cohort (HR = 0.818; 95% CI 0.616–1.085; P = 0.164) or the external validation cohort (HR = 0.716; 95% CI 0.311–1.648; P = 0.432). Chemoradiotherapy was not significantly associated with survival in the SEER cohort (HR = 0.825; 95% CI 0.637–1.068; P = 0.144), although it was associated with improved OS in the external validation cohort (HR = 0.128; 95% CI 0.017–0.951; P = 0.045). However, this result should be interpreted with caution because of the small sample size and imbalanced treatment distribution in the external cohort. Radiotherapy alone was not analyzed separately in the external cohort because only one patient received radiotherapy without chemotherapy. These exploratory findings are summarized in **Table 5**.

**Table 5:** Exploratory analyses of other adjuvant treatment modalities.

Analysis	Cohort	n exposed/ n unexposed	HR (95% CI)	P value
Chemotherapy *vs.* no chemotherapy	SEER cohort	1,179/232	0.818(0.616–1.085)	0.164
	External validation cohort	89/29	0.716(0.311–1.648)	0.432
Chemoradiotherapy *vs.* non-chemoradiotherapy	SEER cohort	1,107/304	0.825(0.637–1.068)	0.144
	External validation cohort	24/94	0.128(0.017–0.951)	0.045

HR, hazard ratio; CI, confidence interval. Surgery alone was defined as no recorded adjuvant radiotherapy or chemotherapy. Chemotherapy alone was defined as chemotherapy without radiotherapy. Radiotherapy alone was defined as radiotherapy without chemotherapy. Chemoradiotherapy was defined as receipt of both radiotherapy and chemotherapy. These.

### SHAP analysis of prognostic contributors

**Figure 8:**
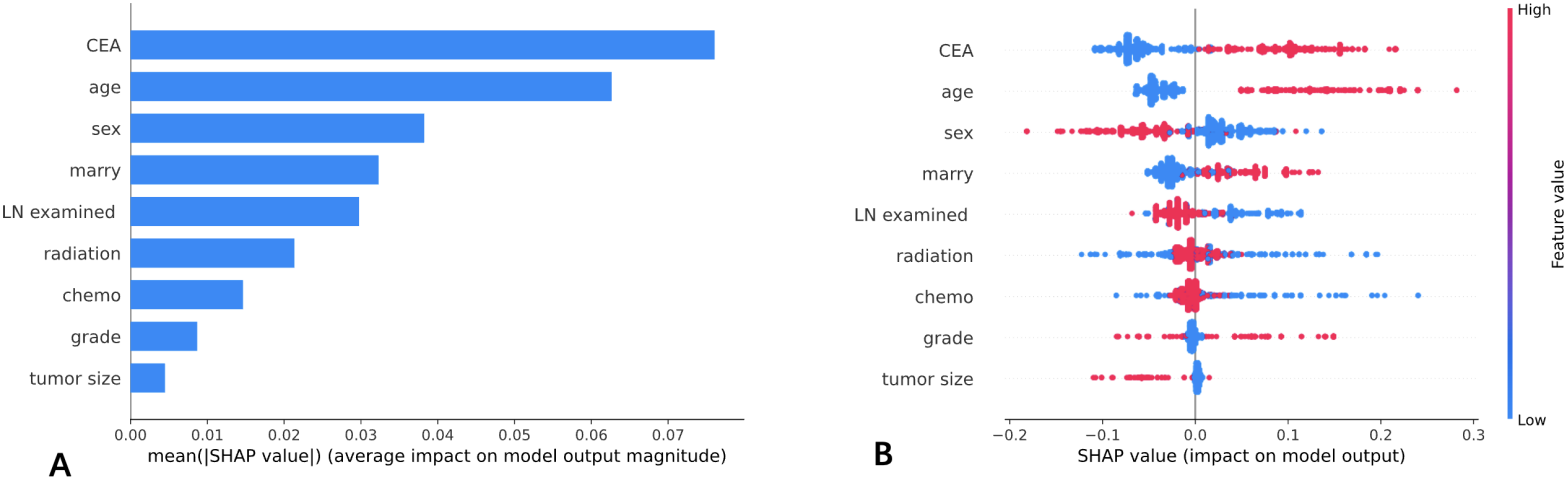
SHAP-based interpretation of the DeepSurv model. **(A)** Global feature importance ranked by mean absolute SHAP value (mean |SHAP|), indicating the average contribution of each predictor to the DeepSurv model output. **(B)** SHAP summary (beeswarm) plot showing the distribution of SHAP values for each feature across individual patients. Positive SHAP values indicate an increased predicted risk, whereas negative values indicate a decreased predicted risk. Dot color represents the feature value (blue = low, red = high); for binary variables, red indicates value = 1 and blue indicates value = 0. SHAP values were computed using the DeepExplainer algorithm with the training dataset as the background reference.

SHAP analysis was applied to improve model interpretability. CEA status was identified as the most influential variable affecting CSS, followed by age, sex, marital status, and other clinicopathological features (**Figure 8**).

## Discussion

### Principal findings

In this study, we developed and externally validated a DeepSurv-based prognostic model to predict CSS in patients with pT3N0 rectal cancer treated with upfront surgery. Using a population-based SEER cohort and an independent real-world cohort, we confirmed that pT3N0 rectal cancer is clinically heterogeneous despite generally favorable outcomes.

Importantly, the model enabled clinically meaningful risk stratification and identified a high-risk subgroup in which adjuvant radiotherapy was associated with significantly improved survival, whereas no benefit was observed among low-risk patients. These findings support a selective, risk-adapted approach to postoperative radiotherapy, consistent with contemporary guideline updates emphasizing individualized decision-making rather than uniform treatment for all stage II patients (15).

### Comparison with prior work

In our previous SEER-based study, we developed a Cox proportional hazard–based risk stratification system and demonstrated that adjuvant radiotherapy improved cancer-specific survival in high-risk pT3N0 patients but not in low-risk patients (16). This work was subsequently cited in the NCCN Clinical Practice Guidelines in Oncology for Rectal Cancer (Version 1.2021) (17), underscoring the clinical relevance of risk-adapted postoperative radiotherapy decision-making in pT3N0 disease. Building on this foundation, we implemented DeepSurv—a Cox-type deep neural network that retains the partial-likelihood formulation while learning a nonlinear risk function—to move beyond the linear predictor imposed by conventional Cox regression and to model potentially complex, higher-order relationships among clinicopathological variables (11).

The present study also differs from our previous Cox-based stratification study in objectives, variable selection, and modeling strategy. The previous study aimed to establish a conventional Cox regression–based risk stratification model and variables were therefore selected within a linear multivariable framework. In contrast, the current study was designed to develop an externally validated DeepSurv-based survival model with potential clinical transportability. Therefore, we prioritized clinicopathological variables that were consistently available, harmonizable, and sufficiently complete across both the SEER cohort and the independent external validation cohort. Although circumferential resection margin and perineural invasion were evaluated in our previous Cox-based study, they were not included in the present model because they were not consistently available or directly comparable between the development and external validation datasets. Including these two variables would have precluded direct external validation using an identical feature set.

Similarly, CEA was retained in the current model despite not entering the final multivariable model in our previous Cox-based analysis. This difference reflects the different modeling frameworks. Traditional Cox models primarily estimate linear independent associations and often rely on statistical variable selection, whereas DeepSurv can incorporate clinically relevant variables and model potential nonlinear or higher-order relationships among predictors. Given its established clinical relevance and broad availability in rectal cancer, CEA was prespecified as a predictor in the DeepSurv model. This decision was further supported by the additional Cox analysis performed in the present study, in which elevated CEA was associated with poorer survival.

Consistent with our results, prior oncology studies have reported improved discrimination for deep learning–based survival models over CoxPH in population-level datasets, including SEER-derived analyses in colon cancer and chondrosarcoma (18, 19). In the comparative analysis, the DeepSurv model showed higher discrimination than the conventional Cox model in the SEER training set and external validation cohort, supporting its potential advantage in capturing nonlinear relationships among clinicopathological variables. This advantage was also visually supported by the time-dependent AUC curves, in which the DeepSurv model generally maintained higher discriminative performance across follow-up time in the training and external validation cohorts. In addition, decision curve analysis suggested that the DeepSurv model provided comparable or higher net benefit than the conventional Cox model across clinically relevant threshold probabilities, particularly in the training and external validation cohorts.

However, the relatively lower discrimination observed in the internal validation cohort should be acknowledged. This may reflect the limited number of outcome events and variability introduced by random splitting of the SEER cohort. More broadly, large comparative studies have shown that the optimal survival algorithm may vary according to population characteristics, endpoint distribution, and validation settings, suggesting the need for external validation and comprehensive performance assessment, including discrimination, calibration, and clinical utility, before clinical translation (20). Against this background, the favorable discrimination and consistent treatment-effect heterogeneity observed across our registry-based and real-world cohorts support the robustness and potential transportability of the proposed DeepSurv-based strategy for postoperative pT3N0 rectal cancer.

### Implications for individualized radiotherapy decision-making

The clinical implication of our findings is straightforward: adjuvant radiotherapy may be prioritized for DeepSurv-defined high-risk patients, while potentially being omitted in low-risk patients. This risk-adapted strategy is clinically relevant given the persistent uncertainty and practice variation regarding adjuvant treatment for pT3N0 rectal cancer in the TME era, as suggested by population-level analyses from the National Cancer Database (NCDB) (21). Notably, other postoperative risk-stratification tools (e.g., nomogram-based models) have similarly emphasized the value of identifying high-risk subgroups to guide adjuvant treatment selection in this clinically heterogeneous population (9).

Real-world postoperative treatment decisions in rectal cancer remain heterogeneous, partly because international guidelines do not provide uniform recommendations for all clinicopathological scenarios. In clinical practice, adjuvant treatment decision is guided by postoperative pathological features, including (y)pT stage, (y)pN stage, margin status, lymphovascular invasion, perineural invasion, and tumor grade. Consistently, Liscu et al. reported that adjuvant chemotherapy use in stage II–III rectal cancer was associated with postoperative pathological characteristics such as (y)pT > 2, (y)pN > 0, and perineural invasion, reflecting variability in treatment selection under inconsistent guideline implementation (22). These findings underscore the need for individualized risk stratification tools for postoperative rectal cancer management.

### Model interpretability and biological plausibility

To address interpretability concerns, we applied SHAP-based explanation to quantify feature contributions at the individual level (23). CEA status emerged as the most influential predictor, which is biologically plausible given accumulating evidence that both pre-/postoperative CEA patterns and postoperative CEA elevation are associated with recurrence and survival outcomes in colorectal/rectal cancer populations (24). Together, interpretability analysis and clinically coherent predictors may facilitate translation of the model into real-world decision support.

### Strengths and limitations

Several strengths of this study should be highlighted. First, this study focused on patients with pathologic T3N0 rectal cancer, a clinically heterogeneous subgroup for whom the optimal use of adjuvant radiotherapy remains controversial. Second, we developed a DeepSurv-based survival model using a population-based SEER cohort and further validated its performance in an independent real-world cohort, thereby improving the clinical relevance and potential generalizability of the model. Third, compared with conventional Cox regression, the DeepSurv model may better capture nonlinear relationships among clinicopathological variables and provide individualized risk stratification. In addition, the model was used not only to predict prognosis but also to explore whether DeepSurv-defined risk groups were associated with differential survival patterns after adjuvant radiotherapy.

Several limitations should also be acknowledged. First, this study was retrospective in nature, and selection bias could not be fully eliminated. Although stratified analyses suggested differential survival patterns according to radiotherapy status, causal inference regarding the effect of adjuvant radiotherapy cannot be definitively established. Second, although external validation was performed using an independent real-world cohort, the sample size of the external validation cohort was relatively limited, and further multicenter validation is warranted. Third, granular treatment and pathological information was unavailable or not consistently comparable across datasets, including radiotherapy dose, target volumes or fields, treatment techniques, treatment adherence, chemotherapy regimens, circumferential resection margin, lymphovascular invasion, and perineural invasion. These factors are important in daily postoperative decision-making and may influence the selection of adjuvant therapy. However, they were not included in the present DeepSurv model to preserve feature consistency for external validation. Therefore, their absence should be considered when interpreting the clinical applicability of the model. Fourth, in response to treatment heterogeneity, we performed additional exploratory analyses according to chemotherapy and chemoradiotherapy status. However, these analyses should be interpreted cautiously because of group imbalance. Thus, the present study should not be interpreted as providing definitive comparative-effectiveness evidence for chemotherapy or chemoradiotherapy. Finally, CSS was used as the endpoint in the SEER cohort, whereas OS was used in the external validation cohort because cancer-specific survival information was unavailable in the real-world cohort. Future prospective multicenter studies incorporating detailed treatment information, molecular features, and standardized survival endpoints are needed before clinical implementation.

Another important direction is the incorporation of emerging prognostic biomarkers. Although the present model was based on routinely available clinicopathological variables, novel markers such as ctDNA, tumor budding, and TLS may provide complementary prognostic information. Postoperative ctDNA may reflect minimal residual disease and help refine recurrence-risk assessment, tumor budding captures invasive tumor behavior, and TLS may reflect the host antitumor immune response. Integration of these biomarkers into future prediction models may further enhance individualized postoperative treatment decision-making in pT3N0 rectal cancer.

## Conclusions

We developed and externally validated a DeepSurv-based risk model for pT3N0 rectal cancer after upfront curative-intent surgery. The model identified clinically relevant treatment-effect heterogeneity, with adjuvant radiotherapy associated with improved survival in high-risk patients but not in low-risk patients. This supports risk-adapted postoperative radiotherapy decision-making and warrants prospective validation.

## Data Availability

The SEER dataset used in this study is not directly shareable by the authors and can be accessed through the SEER Program by registered users. De-identified human subject data supporting the findings of this study will be made available by the authors upon reasonable request.

## References

[1] Siegel RL Giaquinto AN Jemal A . Cancer statistics, 2024. CA Cancer J Clin. (2024) 74:12–49. doi: 10.3322/caac.21820 38230766

[2] vanGijn W Marijnen CA Nagtegaal ID Kranenbarg EM Putter H Wiggers T . Preoperative radiotherapy combined with total mesorectal excision for resectable rectal cancer: 12-year follow-up of the multicentre, randomised controlled TME trial. Lancet Oncol. (2011) 12:575–82. doi: 10.1016/s1470-2045(11)70097-3 21596621

[3] Benson AB Venook AP Al-Hawary MM Azad N Chen YJ Ciombor KK . Rectal cancer, version 2.2022, NCCN clinical practice guidelines in oncology. J Natl Compr Canc Netw. (2022) 20:1139–67. doi: 10.6004/jnccn.2022.0051 36240850

[4] Hofheinz RD Fokas E Benhaim L Price TJ Arnold D Beets-Tan R . Localised rectal cancer: ESMO clinical practice guideline for diagnosis, treatment and follow-up. Ann Oncol. (2025) 36:1007–24. doi: 10.1016/j.annonc.2025.05.528 40412553

[5] You YN Hardiman KM Bafford A Poylin V Francone TD Davis K . The American Society of Colon and Rectal Surgeons Clinical Practice Guidelines for the Management of Rectal Cancer. Dis Colon Rectum. (2020) 63(9):1191–222. doi: 10.1097/DCR.0000000000001762 33216491

[6] Peng LC Milsom J Garrett K Nandakumar G Coplowitz S Parashar B . Surveillance, epidemiology, and end results-based analysis of the impact of preoperative or postoperative radiotherapy on survival outcomes for T3N0 rectal cancer. Cancer Epidemiol. (2014) 38:73–8. doi: 10.1016/j.canep.2013.12.008 24491755

[7] Kucharczyk MJ Bang A Tjong MC Papatheodorou S Fabregas JC . Effectiveness of radiotherapy for local control in T3N0 rectal cancer managed with total mesorectal excision: a meta-analysis. Oncotarget. (2022) 13:1109–19. doi: 10.18632/oncotarget.28280 36251013 PMC9564357

[8] Wu JX Wang Y Chen N Chen LC Bai PG Pan JJ . In the era of total mesorectal excision: adjuvant radiotherapy may be unnecessary for pT3N0 rectal cancer. Radiat Oncol. (2014) 9:159. doi: 10.1186/1748-717x-9-159 25052511 PMC4223727

[9] Zhang C Zhao S Wang X . A prognostic nomogram for T3N0 rectal cancer after total mesorectal excision to help select patients for adjuvant therapy. Front Oncol. (2021) 11:698866. doi: 10.3389/fonc.2021.698866 34900666 PMC8654784

[10] Bhutiani N Peacock O Uppal A You YN Bednarski BK Skibber JM . The current multidisciplinary management of rectal cancer. Ann Gastroenterol Surg. (2024) 8:394–400. doi: 10.1002/ags3.12777 38707228 PMC11066499

[11] Katzman JL Shaham U Cloninger A Bates J Jiang T Kluger Y . DeepSurv: personalized treatment recommender system using a Cox proportional hazards deep neural network. BMC Med Res Methodol. (2018) 18:24. doi: 10.1186/s12874-018-0482-1 29482517 PMC5828433

[12] Sun M Sun J Li M . Deep learning models for predicting the survival of patients with medulloblastoma based on a surveillance, epidemiology, and end results analysis. Sci Rep. (2024) 14:14490. doi: 10.1038/s41598-024-65367-9 38914641 PMC11196279

[13] O'Donnell A Cronin M Moghaddam S Wolsztynski E . A systematic review on machine learning techniques for survival analysis in cancer. Cancer Med. (2025) 14:e71375. doi: 10.1002/cam4.71375 41264402 PMC12633653

[14] Warren JL Klabunde CN Schrag D Bach PB Riley GF . Overview of the SEER-Medicare data: content, research applications, and generalizability to the United States elderly population. Med Care. (2002) 40:Iv–3–18. doi: 10.1097/00005650-200208001-00002 12187163

[15] Benson AB Venook AP Adam M Chang G Chen YJ Ciombor KK . NCCN Guidelines® Insights: Rectal cancer, version 3.2024. J Natl Compr Canc Netw. (2024) 22:366–75. doi: 10.6004/jnccn.2024.0041 39151454

[16] Huang YX Lin YZ Li JL Zhang XQ Tang LR Zhuang QY . Role of postoperative radiotherapy in pT3N0 rectal cancer: A risk-stratification system based on population analyses. Cancer Med. (2019) 8:1024–33. doi: 10.1002/cam4.1991 30714683 PMC6434337

[17] Network, N.C.C . NCCN clinical practice guidelines in oncology (NCCN Guidelines®): Rectal cancer. Version 1.2021 (2021). Available online at: https://www.nccn.org (Accessed February 6, 2026).

[18] Qu Z Wang Y Guo D He G Sui C Duan Y . Comparison of deep learning models to traditional Cox regression in predicting survival of colon cancer: Based on the SEER database. J Gastroenterol Hepatol. (2024) 39:1816–26. doi: 10.1111/jgh.16598 38725241

[19] Yan L Gao N Ai F Zhao Y Kang Y Chen J . Deep learning models for predicting the survival of patients with chondrosarcoma based on a surveillance, epidemiology, and end results analysis. Front Oncol. (2022) 12:967758. doi: 10.3389/fonc.2022.967758 36072795 PMC9442032

[20] Yuan L Wang L Gao J Chen X Wang H Tan WS . OncoE25: an AI model for predicting postoperative prognosis in early-onset stage I-III colon and rectal cancer—a population-based study using SEER with dual-center cohort validation. J Transl Med. (2025) 23:695. doi: 10.1186/s12967-025-06663-4 40545536 PMC12183820

[21] Quinn TJ Rajagopalan MS Gill B Mehdiabadi SM Kabolizadeh P . Patterns of care and outcomes for adjuvant treatment of pT3N0 rectal cancer using the National Cancer Database. J Gastrointest Oncol. (2020) 11:1–12. doi: 10.21037/jgo.2019.10.02 32175100 PMC7052766

[22] Liscu HD Liscu BR Mitre R Anghel IV Antone-Iordache IL Balan A . The conditioning of adjuvant chemotherapy for stage II and III rectal cancer determined by postoperative pathological characteristics in Romania. Med (Kaunas). (2023) 59(7):1224. doi: 10.3390/medicina59071224 37512037 PMC10384917

[23] Lundberg SM Lee S-I . A unified approach to interpreting model predictions. In: Proceedings of the 31st International Conference on Neural Information Processing Systems. Curran Associates Inc, Long Beach, California, USA (2017). p. 4768–77.

[24] Nakamura Y Shida D Tanabe T Takamizawa Y Imaizumi J Ahiko Y . Prognostic impact of preoperatively elevated and postoperatively normalized carcinoembryonic antigen levels following curative resection of stage I-III rectal cancer. Cancer Med. (2020) 9:653–62. doi: 10.1002/cam4.2758 31799750 PMC6970051

