# Correction: Risk stratification for adjuvant radiotherapy in pathologic T3N0 rectal cancer using a DeepSurv-based survival model

**DOI:** 10.3389/fonc.2026.1901747

**Published:** 2026-06-12

**Authors:** Yunxia Huang, Yanzong Lin, Qingyang Zhuang, Xinyuan Wu, Qiping Fan, Junxin Wu

**Affiliations:** 1 Department of Radiation Oncology, Clinical Oncology School of Fujian Medical University, Fujian Cancer Hospital, Fuzhou, China; 2 Department of Radiation Oncology, The First Affiliated Hospital of Xiamen University, School of Medicine, Xiamen University, Teaching Hospital of Fujian Medical University, Xiamen, China; 3 Department of General Surgery, The First Affiliated Hospital of Xiamen University, School of Medicine, Xiamen University, Teaching Hospital of Fujian Medical University, Xiamen, China; 4 Department of Urology, The First Clinical Medical College of Southern Medical University, Guangzhou, China; 5 Department of Public Health Sciences, Clemson University, Clemson, SC, United States

**Keywords:** adjuvant radiotherapy, DeepSurv, pT3N0 rectal cancer, risk stratification, survival model

The funder “Fujian Province Gastrointestinal, Respiratory, Genitourinary Malignant Tumor Radiotherapy Radiation and Treatment Clinical Medical Research Center (2021Y2014)” was erroneously omitted.

The original version of this article has been updated.

